# Enhanced Effect of Polyethyleneimine-Modified Graphene Oxide and Simvastatin on Osteogenic Differentiation of Murine Bone Marrow-Derived Mesenchymal Stem Cells

**DOI:** 10.3390/biomedicines9050501

**Published:** 2021-05-02

**Authors:** Jun-Sung Oh, Jeong-Sun Park, Eun-Jung Lee

**Affiliations:** Department of Nano-biomedical Science, Dankook University, Cheonan 31116, Korea; gda4101@dankook.ac.kr (J.-S.O.); kimh1811@gmail.com (J.-S.P.)

**Keywords:** simvastatin, graphene oxide, polyethylenimine, mesenchymal stem cells, osteogenic differentiation

## Abstract

Statin derivatives traditionally have been used for the treatment of hyperlipidemia, but recent studies have shown their ability to regulate bone metabolism and promote bone growth. In this study, simvastatin (Sim), a new therapeutic candidate for bone regeneration, was combined with graphene oxide (GO), which has recently attracted much interest as a drug delivery method, to produce a compound substance effective for bone regeneration. To create a stable and homogenous complex with Sim, GO was modified with polyethylenimine, and the effect of modification was analyzed using Fourier transform infrared spectroscopy, zeta potential, and cytotoxicity testing. More specifically, the osteogenic differentiation potential expected by the combination of the two effective materials for osteogenic differentiation, GO and Sim, was evaluated in mesenchymal stem cells. Compared with control groups with GO and Sim used separately, the GO/Sim complex showed excellent osteogenic differentiation properties, with especially enhanced effects in the complex containing < 1 μM Sim.

## 1. Introduction

A drug delivery system should be biocompatible, mechanically strong (physical stable), and capable of high-volume drug loading, easy-to-control drug conjugation, and release [1,2,3,4]. The carriers in the drug delivery system (DDS) can prolong the duration of drug efficacy, reduce drug metabolism, and improve the delivery of the drug to the target site [5]. Research on more advanced drug delivery technology and new carriers as therapeutic candidates are continuously being pursued for ideal therapeutic outcomes, and two-dimensional (2D) graphene-based materials have recently attracted increasing interest [6,7].

Owing to its 2D structure, graphene oxide (GO) has a high drug loading per mass ratio for drug delivery [8,9]. In particular, its various oxygen-containing groups (C=O, COOH, OH, C-O-C) ensure good suspension stability in water solutions and ready modification and control of the surface for attachment of proteins or drug molecules [10,11,12]. Thus, this material has been widely researched in DDS [13,14]. The biologically stable concentration of GO has been reported to be approximately ≤20 μg/mL [15]. It indicates superior biocompatibility and greater potential for bio-applications compared with other carbon-based nanomaterials such as carbon nanotubes, which have a limited usable concentration of <3 μg/mL in biological systems because of cytotoxicity-related concerns [16]. Moreover, recent studies have indicated the promotion of growth, proliferation, and differentiation of osteoblasts by GO; it has been gaining increasing attention as a promising therapeutic candidate for osseous tissue regeneration [17,18].

GO has previously been incorporated with drugs for the development of effective therapeutic substances. The drugs include bone morphogenetic protein-2 (BMP-2), alendronate, dexamethasone, etc. All of the drugs showed that GO plays a synergistic role in bone differentiation [19,20,21]. In this study, simvastatin (Sim), a new drug candidate in the bone regeneration field, was combined with GO to produce a compound that may be effective for bone regeneration. Sim has been traditionally used to treat hyperlipidemia, but recent studies have reported its ability to regulate BMP-2, vascular endothelial growth factor, and bone metabolism while promoting bone formation [22,23,24,25]. Although nanoparticle-based carriers for Sim have been previously studied [26], regeneration of hard tissue requires a higher dose than otherwise required for the treatment of hyperlipidemia or neuronal regeneration. In order to induce the efficacy of Sim for hard tissue engineering, drug carriers with higher loading capacity or in combination with substances such as osteogenic boosters of Sim that can facilitate appropriate effect on bone repair using a small amount of Sim, should be considered.

In this study, a therapeutic medicine combining 2D graphene and Sim was fabricated to support the osteogenic function of Sim, and the synergistic effect of the complex for bone regeneration was assessed utilizing mesenchymal stem cells (MSCs).

## 2. Materials and Methods

### 2.1. Materials

The following materials were purchased from various companies. Graphene oxide (Graphene super market, Reading, MA, USA), N-(3-Dimethylaminopropyl)-N′-ethylcarbodiimide hydrochloride (EDC, Sigma-Aldrich, St. Louis, MO, USA), Polyethyleneimine (PEI, ~Mw 25k, Sigma-Aldrich, St. Louis, MO, USA), dialysis membrane (pore size 30k), simvastatin (Sigma-Aldrich, St. Louis, MO, USA), and fetal bovine serum (FBS, Hyclone, Waltham, MA, USA). penicillin/streptomycin (P/S, Gibco, Waltham, MA, USA), Dulbecco’s modified Eagle medium (DMEM, Welgen, Gyeongsan-si, Korea), β-glycerophosphate (Sigma-Aldrich, St. Louis, MO, USA), Dexamethasone (Sigma-Aldrich, St. Louis, MO, USA), l-ascorbic acid (Sigma-Aldrich, St. Louis, MO, USA), MTT assay (Thermo Fisher Scientific, Waltham, MA, USA), paraformaldehyde (PFA, Sigma-Aldrich, St. Louis, MO, USA), Phalloidin Alexa 488 (Thermo Fisher Scientific, Waltham, MA, USA), ALP detection kit (Biovision, Milpitas, CA, USA), Bradford assay buffer (Thermo Fisher Scientific, Waltham, MA, USA), Alizarin Red S (ARS, Sigma-Aldrich, St. Louis, MO, USA), cDNA Synthesis Kit (Nanohelix, Daejeon, Korea), and primers including RUNX2, OPN, and OCN (Bioneer, Daejeon, Korea).

### 2.2. Synthesis of Go-Polyethylenimine (GP)/Simvastatin (Sim) Complexes

GO (1 mg/mL) suspension was prepared at pH 1 by adjusting its pH with HCl, and 386 mg of ethyl (dimethylaminopropyl) carbodiimide (EDC) was added in and stirred for 20 min. Then, 115 mg N-hydroxysuccinimide (NHS) was added and stirred for an additional hour. Afterward, 200 mg PEI was dissolved in deionized water and mixed with the prepared GO solution for a reaction duration of 1 day. GO-PEI solution was purified by removing unreacted residues by dialysis via dialysis membrane for 2 days, and the final GO-PEI (GP) product in powder formulation was obtained by lyophilization. On the basis of previously performed cytotoxicity testing, the concentration of GP for this study was set at 10 μg/mL [27], and GP/Sim (GS) complex was produced by allowing approximately 0.25–2 μM Sim to attach to GP at room temperature for 3 h, forming complexes. Although the data were not presented in this manuscript, we previously investigated the adsorption rate of Sim at different concentrations. We incorporated 100% and 84% Sim at 0.25–1 μM and 2 μM, respectively, into GP. The obtained complexes were labeled according to the Sim concentration as follows: GS1 (0.25 μM Sim), GS2 (0.5 μM Sim), GS3 (1 μM Sim), and GS4 (2 μM Sim). Individual GP and 1 μM Sim were used as control groups for all experiments.

### 2.3. Property Investigations

The properties of GP, PEI, and GO were analyzed by Fourier transform infrared spectroscopy (FT-IR Agilent, Santa Clara, CA, USA). The zeta potential of materials was measured at 100 μg/mL suspension in water using a Zetasizer instrument (Nano-ZS, Malvern, WR, UK).

### 2.4. Tests for Cell Viability

MSCs were isolated from mouse (5 weeks, male) bone marrow harvested from the tibia and femoral marrow compartments, then cultured in general cell media, utilizing Dulbecco’s Modified Eagle Medium supplemented with 10% fetal bovine serum and 1% penicillin–streptomycin at 37 °C, with 5% CO_2_, and at 90% humidity. Cells for cell adhesion study were prepared by dispensing 1 × 10^4^ cells/mL in each of 24 well plates. Prepared materials were added after 24 h. Then, the MSCs were fixed on days 1 and 3 by adding 4% paraformaldehyde (PFA) and treating with Triton X-100 for 10 min. They were then reacted with 200 μL Alexa 488 phalloidin solution for 1 h in each well. Once the reactions were completed, samples were rinsed with phosphate buffer saline (PBS) and monitored using confocal laser scanning microscopy (CLSM, Zeiss, GER). MSCs at a concentration of 3 × 10^3^ cells/well were seeded on 96 well plates and cultured for 1, 3, and 7 days in general cell media for the confirmation of cell proliferation. On days 1, 3, and 7, samples were rinsed with PBS and reacted with MTT for 4 h. Then, they were treated with sodium dodecyl sulfate for 4 h and measured using a microplate reader (Spectramax) at 570 nm wavelength.

### 2.5. Alkaline Phosphatase (ALP) Activity Assay

Early stage bone differentiation indicator, or ALP activity, was examined using the ALP detection kit. MSCs at a concentration of 1 × 10^4^ cells/mL were seeded and cultured in the presence of test materials for 7 and 14 days in bone differentiation cell media. Bone differentiation culture media was prepared by adding 50 μg/mL ascorbic acid, 10 mM β-glycerophosphate, and 100 nM dexamethasone into the general cell culture media. Adhered cells were rinsed with PBS thrice and lysed with Triton X-100 for 5 min. The protein quantity was determined by the Bradford assay kit, and an equal amount of protein was compensated into samples. Samples were reacted with para-nitrophenyl phosphate (pNPP) and measured at 405 nm wavelength.

### 2.6. Alizarin Red S (ARS) Staining

MSCs were cultured in bone differentiation media, and the bone differentiation was confirmed by Alizarin Red S (ARS) staining. After 21 days of culture, cells were fixed with 4% PFA for 15 min and reacted with 2% ARS for 10 min. For quantitative analysis, ARS staining was extracted with 10% acetic acid for 30 min and neutralized by 10% ammonium hydroxide and then measured at 405 nm wavelength.

### 2.7. Quantitative Real Time Polymerase Chain Reaction

Using MSCs cultured in the same media condition as the ALP test, bone differentiation determined by gene expression was measured by quantitative real time polymerase chain reaction (qRT-PCR) after 7 and 14 days of cell culture. Cell pellets obtained from cell cultures were treated with Trizol reagent to extract its RNA, and cDNA was synthesized using the cDNA synthesis kit. Glyceraldehyde 3-phosphate dehydrogenase (GAPDH) mRNA was used as an internal control to check PCR amplification. Runt-related transcription factor 2 (RUNX2), osteopontin (OPN), and osteocalcin (OCN) were used as primers. The thermal profile for PCR was 50 °C for 2 min, 95 °C for 10 min, and 40 cycles performed at 95 °C for 15 s, followed by 60 °C for 1 min. Finally, cycle threshold values were used to evaluate the difference in gene expression among the groups.

### 2.8. Statistical Analysis

All quantitative results were collected in at least 3 replicates from each test group. The statistical analyses were performed using *t*-test, and comparisons between groups were analyzed by the one-way analysis of variance test. The differences with a *p* value < 0.01 were considered statistically significant (** *p* < 0.01, *** *p* < 0.001).

## 3. Results

### 3.1. Simvastatin Immobilization by PEI Modified-GO (GP)

**Figure 1:**
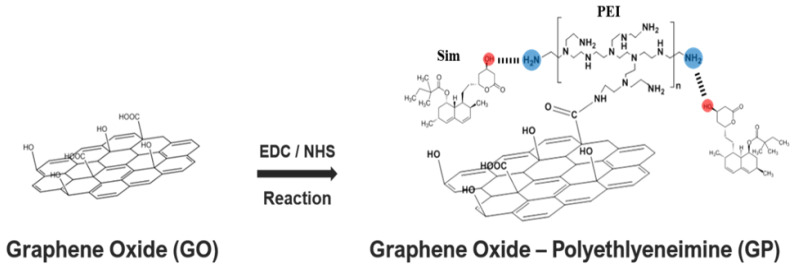
Schematic representation of the formation of polyethyleneimine-modified graphene oxide/simvastatin complexes. EDC: ethyl (dimethylaminopropyl) carbodiimide; NHS: N-hydroxysuccinimide. Modified from [29].

Polyethyleneimine (PEI) has drawn attention as a carrier agent for biomolecule immobilization in a number of biosystems, because it has a high concentration of amino groups [28]. PEI as a physical glue useful for bonding biomolecules via physical interaction was modified on GO through EDC/NHS conjugation chemistry (Figure 1). The OH and COO groups in GO easily facilitate complexation with and delivery of positively charged drug molecules by electric attraction, but they pose limitations for negatively charged drugs, such as Sim. However, for a DDS utilizing the advantage of the 2D planar structure of GO, various surface modification techniques have been used for the incorporation of drugs to overcome these limitations. In this study, the GO surface charge was reversed to positive by modifying PEI on the surface for complexation with Sim, resulting in the stable and effective incorporation of GO and Sim.

### 3.2. Chemical Bond Identification

**Figure 2:**
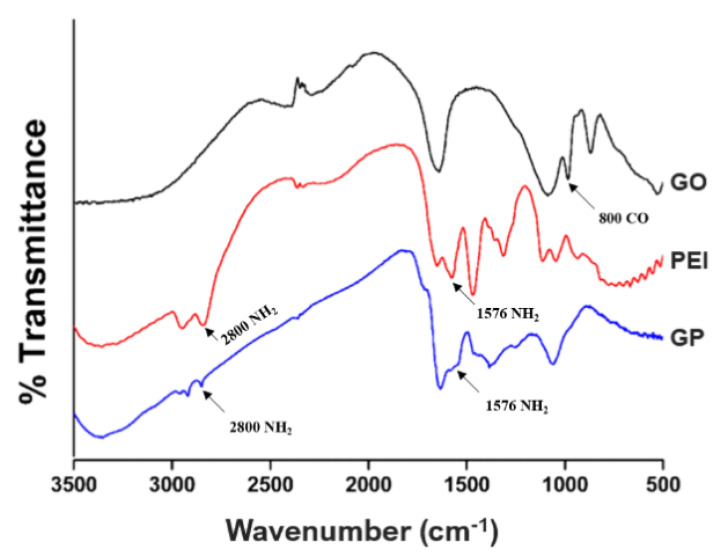
Fourier transform infrared spectrum of graphene oxide (GO), polyethyleneimine (PEI), and GO-PEI product. Modified from [29].

FT-IR analysis was performed to confirm whether PEI was conjugated properly on the GO surface or any undesired products were generated (Figure 2). Peaks at 2800 cm^−1^ and 1576 cm^−1^ (amine groups) were observed from surface-processed GP, which are PEI’s characteristic peaks [30,31]. On a side note, the intensity of amine group peaks was weaker in GP than in individual PEI. Furthermore, the 800 cm^−1^ peak of C-O (or COO or OH) as seen in the GO spectrum was not observed in the GP spectrum [32]. This indicated that a bond had formed between the COOH group in GO and the amine group in PEI when the surface of GO was modified with PEI. In conclusion, GP was successfully produced by EDC/NHS reaction without the formation of a secondary phase.

### 3.3. Zeta Potential Determination

**Figure 3:**
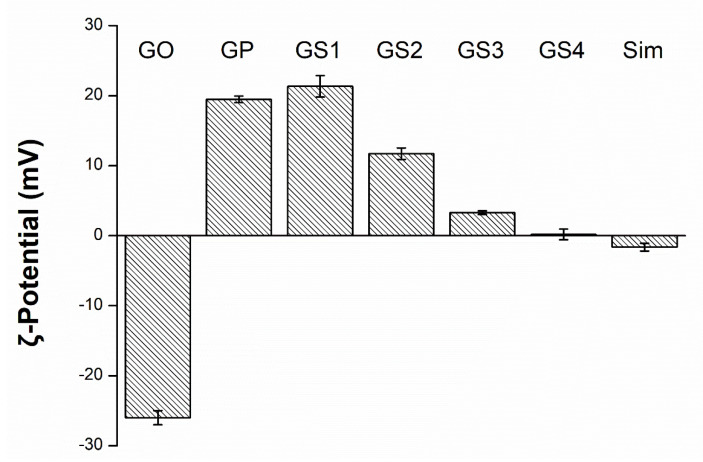
The electrical characteristics of graphene oxide (GO), GO-polyethyleneimine (GP), simvastatin (Sim), and GP/Sim (GS1–GS4) measured with zeta potential. Modified from [29].

The investigation of zeta potential is a significant characterization of nanocrystals to assess the surface charge, which can be an indicator for homogeneity in water solution and an important property for uniform and stable binding with drugs [33]. In this study, we also measured the potential of the GP/Sim complex to evaluate the conjugation efficiency of graphene with drugs with different potential values. The evaluated zeta potential of each material in this study is shown in Figure 3. It was confirmed that initially negatively charged GO became positively charged after conjugation with PEI. The zeta potential of GS, which was complexed with the weakly negatively charged Sim (about −4 mV) became close to negative as the concentration of Sim increased. Therefore, stable PEI treatment of GO and successful complexation with Sim were confirmed.

### 3.4. Cytotoxicity and Proliferation

**Figure 4:**
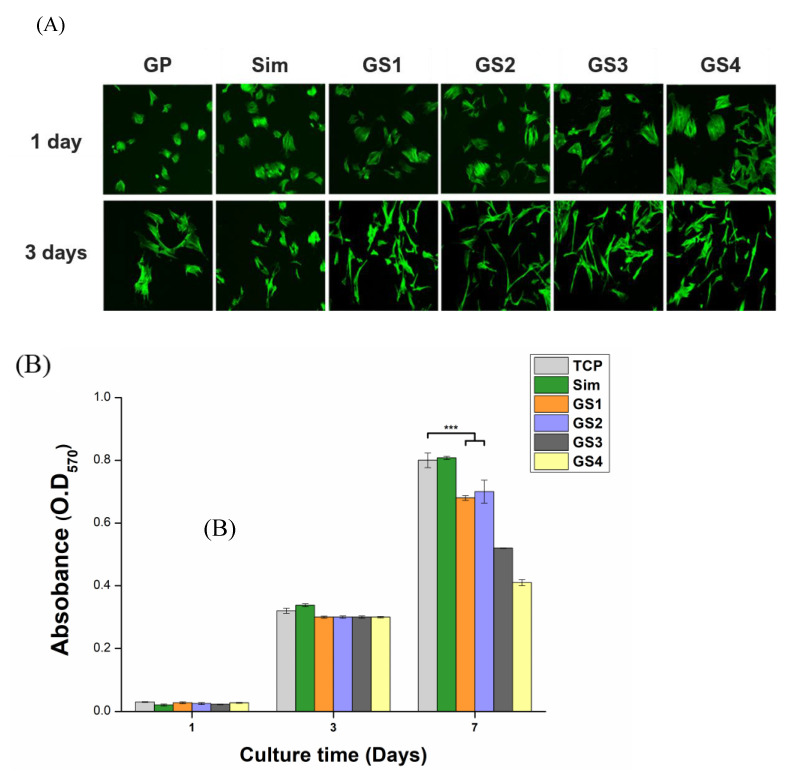
In vitro cell test results. Cell attachment revealed by CLSM (**A**). Proliferation of MSCs for 7 days of culturing (**B**). CLSM images were taken at 100× magnification. Error bars represent +/− standard deviations (*n* ≥ 3). *** *p* < 0.001. Modified from [29].

Although GO can be considered as a drug-eluting carrier, the present study was focused on the evaluation of its cellular performance as a drug-combined inorganic medicine rather than its drug release profile. Various cell tests were performed in MSCs to evaluate the biological stability and function of the obtained products. According to the literature, Sim showed the best cell proliferation and bone differentiation ability at 1 μM [34,35,36]. Thus, in the current study, we developed GS complexes with the concentration of Sim at around 1 μM to evaluate the cellular responses. First, the cytotoxicity of GP and GS was examined by monitoring the change in cell adhesion and proliferation. Cells that were visible in green by Phalloidin Alexa 488 were appropriate for observing changes in attachment behaviors depending on the materials and culture time. CLSM images of cell adhesion in MSCs cultured in the presence of test materials are shown in Figure 4A. The adhesion behavior of cells cultured in tissue culture plate (TCP) were similar to that under the GP condition (not shown here). Images showed that, in all test materials, cells kept their shape without showing cell death. However, cells cultured in the presence of GS showed relatively greater adhesion surface area compared with those cultured in the presence of GP or Sim on day 1, whereas they appeared thinner and more elongated on day 3. The observation of cell morphology provides important evidence to predict or analyze the growth and differentiation of stem cells. Elongation of cell shape is often considered an indicator of bone differentiation [37]. Therefore, GS complexation can be expected to promote bone differentiation. MTT assay results showed that consistent cell proliferation occurred for 7 days without any sign of cytotoxicity (Figure 4B). Although no significant difference among test materials was observed for the first 3 days, they showed a significant difference on day 7. TCP and Sim showed the highest cell proliferation efficiency, whereas GS1 and GS2 showed similar efficiency and GS3 or higher exhibited relatively slower proliferation behaviors. As the overall cellular responses showed a tendency to decrease from GS3, subsequent bone differentiation studies evaluated samples up to GS3, excluding GS4.

### 3.5. ALP Activity

**Figure 5:**
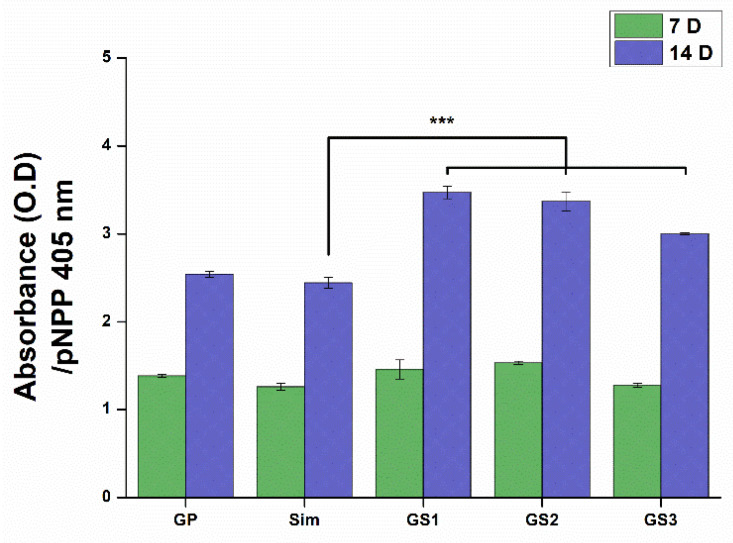
Alkaline phosphatase activity of mesenchymal stem cells on graphene oxide-polyethyleneimine-simvastatin complexes with different simvastatin content after 7 days and 14 days of culturing. Error bars represent +/− standard deviations (*n* ≥ 3). *** *p* < 0.001. Modified from [29].

ALP is an enzyme presenting on the surface of cell membranes, and is expressed highly during early stages of bone formation. Thus, ALP activity in cells can be used to evaluate bone differentiation [38]. ALP activity from each test material is shown in Figure 5. ALP activity from cells cultured for 7 days did not show recognizable differences, but that from cells cultured for 14 days showed that ALP activity was higher in the GS complex than in GP and Sim alone. More specifically, GS1 and GS2 showed a highly statistically significant increase in ALP activity, while GS3 containing 1 μM Sim revealed a reduction in the level of ALP. This indicated that GP/Sim combination effectively stimulates the initial osteogenic differentiation of MSCs.

### 3.6. Mineralization of Stem Cells

**Figure 6:**
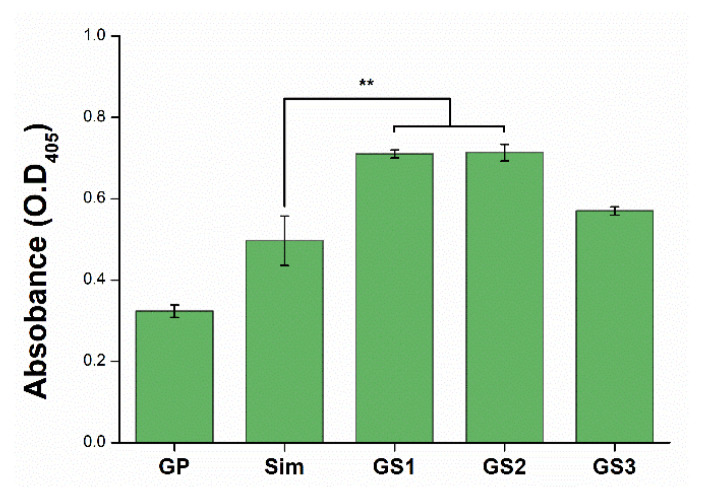
Alizarin Red S assay result for mesenchymal stem cells after 21 days of culturing. Error bars represent +/− standard deviations (*n* ≥ 3). ** *p* < 0.01. Modified from [29].

ARS staining is a method used to analyze the mineralization of cells and the extracellular matrix, which is an indicator of the last stage of bone differentiation [39]. After a cell culture of 21 days, calcium deposits in cells were quantified by staining with ARS reagent (Figure 6). Cells treated with GS complexes exhibited higher bone differentiation than those treated with GP or Sim alone, which are known to promote bone differentiation (Figure 6). More specifically, the promoted effect was observed in GS1 and GS2, whereas GS3 containing 1 μM Sim showed a relatively lower effect. The combination of GP and Sim dramatically augmented osteogenic differentiation using bone differentiation cell media, compared to that of a single material (GP, Sim).

### 3.7. Osteogenic Gene Expression

**Figure 7:**
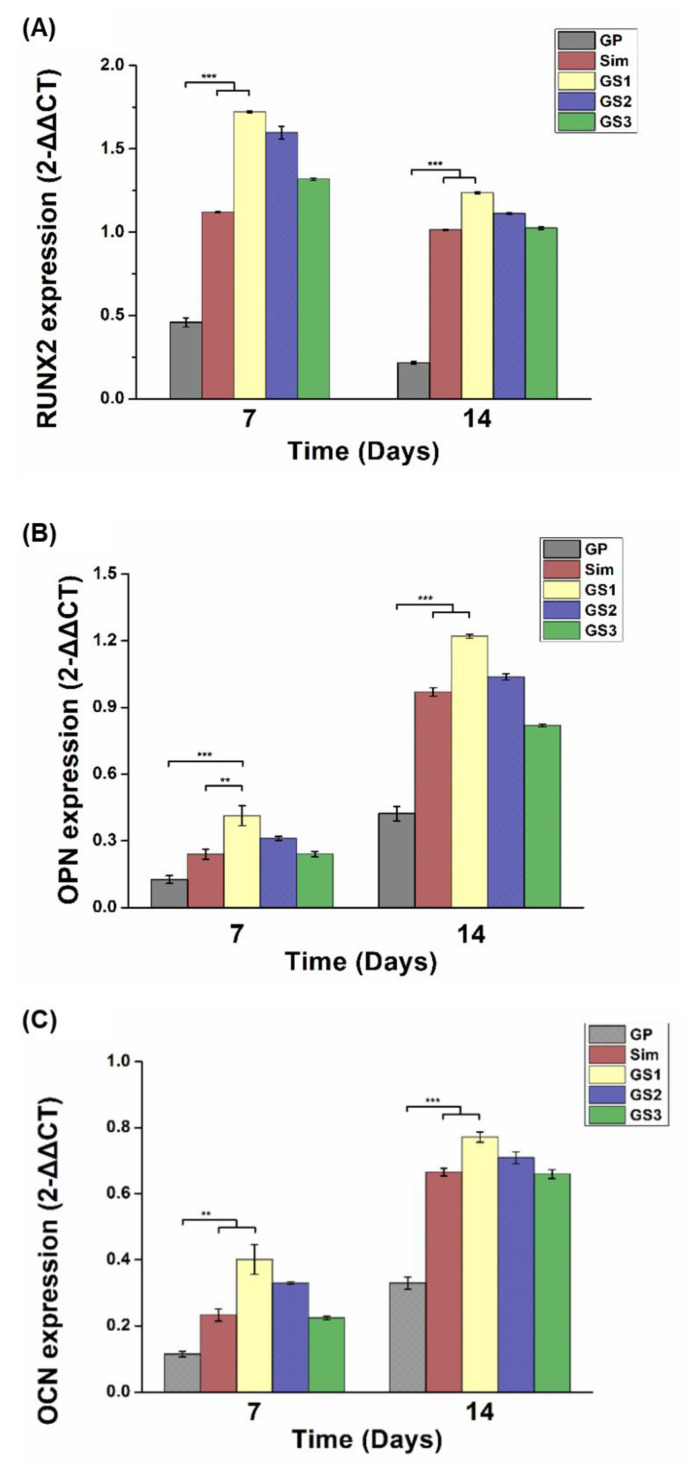
Real-time polymerase chain reaction following in vitro mesenchymal stem cell culturing with culture plate (control), and concentration of graphene oxide-polyethyleneimine-simvastatin complex treatment after 7 and 14 days of culturing. Early marker runt-related transcription factor 2 (**A**) and late markers osteopontin (**B**) and osteocalcin (**C**). Error bars represent +/− standard deviations (*n* ≥ 3). ** *p* < 0.01 and *** *p* < 0.001. Modified from [29].

The enhanced effect of GS on bone differentiation was further evaluated through the cellular gene expression efficiency via qRT-PCR. GO is known to have a favorable effect on cell proliferation and differentiation because it has a large number of hydrophilic functional groups that can induce adhesion of proteins related to cell attachment. In particular, the high stiffness and osteoconductivity of GO support the promotion of osteogenic differentiation of bone-related cells [40,41]. The GO in GP/Sim complexes has the roles of expanding the biomedical application of GP/Sim as a matrix that can form coatings, membranes, and scaffolds as well as serving as an osteoconductive supporter. Simvastatin is also well-known for stimulating the BMP-Smad signaling pathway and VEGF signaling pathway to up-regulate bone-related genes. qRT-PCR is a polymerase chain reaction–based technique; the products of qRT-PCR amplification can be monitored in real time, making this technique very useful to the study of cell differentiation through the quantification of DNA [42]. In this study, the expression levels of bone differentiation markers were examined after cell culture durations of 1 and 2 weeks. The bone differentiation markers used in the study were RUNX2, OPN, and OCN, which are representative markers for the early, middle, and later stages of bone differentiation, respectively [43,44]. GS showed a higher level of gene expression for all markers than GP (Figure 7). When looking at the differences dependent on culture duration, the early-stage marker, RUNX2, was higher in the cells cultured for 7 days than in those cultured for 14 days, whereas the middle- and later-stage markers were higher after 14 days than after 7 days. Among the GSs, GS1 complexed with 0.25 μM Sim showed the strongest level of gene expression for bone differentiation. This value was over 3–4 times higher than GP and indicated at least 20% greater bone differentiation compared with Sim alone. Interestingly, GS3 complexed with 1 μM Sim exhibited very similar behavior to the one treated with Sim alone at 1 μM in all gene expressions. The *t*-test was conducted for each data pair. All means of the specimens showed significant differences.

These results demonstrated that GSs certainly induce an enhanced effect on bone differentiation, and that the synergies are reduced by combining GP with more than 1 μM of Sim. This work was meaningful in providing information on the optimal concentration with the best bone differentiation capability when GO and Sim are combined. Furthermore, the developed GP/Sim complex could be applied in the form of coatings, nanofibers, membranes, films, scaffolds, etc., as a platform for drug-converged, inorganic-based medicine. Therefore, we expect that this study will be of great interest to researchers in the field of hard tissue engineering.

## 4. Conclusions

In this study, a compound effective in promoting bone differentiation was produced by incorporating graphene with a statin derivative (Sim), an emerging drug candidate for bone regeneration, to evaluate their enhanced effects. The GO was modified with PEI to allow for complexation with negatively charged Sim. Zeta potential measurements confirmed a stable bond formation between GP and Sim via electrical attraction. The promoted effect of the GO/Sim complex on osteogenic differentiation and osseous formation was evaluated by cellular adhesion on stem cells as well as cellular proliferation and differentiation behaviors. In conclusion, GS complexes showed superior bone differentiation properties compared with either GP or Sim alone, whereas GS1 complexed with 0.25 μM Sim showed the greatest improved effect.
