# Correction: Oh et al. Enhanced Effect of Polyethyleneimine-Modified Graphene Oxide and Simvastatin on Osteogenic Differentiation of Murine Bone Marrow-Derived Mesenchymal Stem Cells. *Biomedicines* 2021, *9*, 501

**DOI:** 10.3390/biomedicines10081802

**Published:** 2022-07-27

**Authors:** Jun-Sung Oh, Jeong-Sun Park, Eun-Jung Lee

**Affiliations:** Department of Nano-biomedical Science, Dankook University, Cheonan 31116, Korea; gda4101@dankook.ac.kr (J.-S.O.); kimh1811@gmail.com (J.-S.P.)

## Addition of an Author

**Jeong-Sun Park** was not included as an author in the original publication [1]. The corrected Author Contributions Statement appears here.

**Author Contributions**: Conceptualization, E.-J.L.; methodology, E.-J.L. and J.-S.O.; investigation, E.-J.L., J.-S.P. and J.-S.O.; validation, J.-S.P. and J.-S.O.; writing—original draft preparation, E.-J.L. and J.-S.O.; writing—review and editing, E.-J.L. and J.-S.O.; supervision, E.-J.L.; project administration, E.-J.L.; funding acquisition, E.-J.L. All authors have read and agreed to the published version of the manuscript.

## Missing Citation

In the original publication [1], **Park** (**2018**) was not cited. The citation has now been inserted in the legends of figures and references’ order has also been adjusted.

29. Park, J.-S. Carbon-Based Graphene as a Drug Carrier for Use in Tissue Engineering. Master’s Thesis, Dankook University, Cheonan, Korea, 8 January 2018. (In Korean)

## Figure Legend

In the original publication [1], there was a mistake in the legends of all figures. **This study is based on Jeong-Sun Park’s Master thesis [29], so the Master thesis has to be cited in the legends of those figures**.

**Figure 1. Schematic representation of the formation of polyethyleneimine-modified graphene oxide/simvastatin complexes. EDC: ethyl (dimethylaminopropyl) carbodiimide; NHS: N-hydroxysuccinimide. Modified from [29].**

**Figure 2. Fourier transform infrared spectrum of graphene oxide (GO), polyethyleneimine (PEI), and GO-PEI product. Modified from [29].**

**Figure 3. The electrical characteristics of graphene oxide (GO), GO-polyethyleneimine (GP), simvastatin (Sim), and GP/Sim (GS1–GS4) measured with zeta potential. Modified from [29].**

Figure 4. In vitro cell test results. Cell attachment revealed by CLSM (A). Proliferation of MSCs for 7 days of culturing (B). CLSM images were taken at 100× magnification. Error bars represent +/− standard deviations (*n* ≥ 3). *** *p* < 0.001. Modified from [29].

Figure 5. Alkaline phosphatase activity of mesenchymal stem cells on graphene oxide–polyethyleneimine–simvastatin complexes with different simvastatin content after 7 days and 14 days of culturing. Error bars represent +/− standard deviations (*n* ≥ 3). *** *p* < 0.001. Modified from [29].

Figure 6. Alizarin Red S assay result for mesenchymal stem cells after 21 days of culturing. Error bars represent +/− standard deviations (*n* ≥ 3). ** *p* < 0.01. Modified from [29].

Figure 7. Real-time polymerase chain reaction following in vitro mesenchymal stem cell culturing with culture plate (control), and concentration of graphene oxide–polyethyleneimine–simvastatin complex treatment after 7 and 14 days of culturing. Early marker runt-related transcription factor 2 (A) and late markers osteopontin (B) and osteocalcin (C). Error bars represent +/− standard deviations (*n* ≥ 3). ** *p* < 0.01 and *** *p* < 0.001. Modified from [29].

The authors apologize for any inconvenience caused and state that the scientific conclusions are unaffected. This correction was approved by the Academic Editor. The original publication has also been updated.

## References

[1] Oh J.-S., Park J.-S., Lee E.-J. (2021). Enhanced Effect of Polyethyleneimine-Modified Graphene Oxide and Simvastatin on Osteogenic Differentiation of Murine Bone Marrow-Derived Mesenchymal Stem Cells. Biomedicines.

